# DAPK-1 as a Potential Early Marker for Malignant Transformation Risk of Oral Lichen Planus

**DOI:** 10.7759/cureus.71714

**Published:** 2024-10-17

**Authors:** Petros Papadopoulos, Vasileios Zisis, Dimitrios Andreadis, Konstantinos Poulopoulos, Dimitrios Parlitsis, Konstantinos Paraskevopoulos, Pinelopi A Anastasiadou, Eleftherios Anagnostou, Konstantinos Vahtsevanos, Athanasios Poulopoulos

**Affiliations:** 1 Oral Medicine and Pathology, Aristotle University of Thessaloniki, Thessaloniki, GRC; 2 Oral and Maxillofacial Surgery, Papanikolaou Hospital, Aristotle University of Thessaloniki, Thessaloniki, GRC; 3 Oral and Maxillofacial Surgery, Aristotle University of Thessaloniki, Thessaloniki, GRC

**Keywords:** dapk, erosive oral lichen planus, immunohistochemistry staining, oral leukoplakia, oral lichen planus, oscc

## Abstract

Introduction

Oral lichen planus (OLP) comprises a chronic inflammatory autoimmune disease observed in the oral cavity. It most commonly manifests as white papules arranged confluently, drawing a picture of white lines in the form of a network (reticular form). It may emerge in other forms as well. In our study, the erosive form presents the most clinical interest. Among the biomarkers that participate in the tumorigenesis process, DAPK-1 seems promising, rendering its study necessary. This study focuses on the investigation of the presence of the DAPK-1 using immunohistochemistry in OLP compared to oral squamous cell carcinoma (OSCC), oral leukoplakia (OL), a well-established oral potentially malignant disorder, and normal oral epithelium to evaluate its possible role as an early predictor of the possibility of malignant transformation risk of OLP lesions.

Methods

To monitor the expression profile of the tumor suppressor protein DAPK-1, an immunohistochemical detection took place in 18 samples of OLP (reticular and erosive type), in 22 OSCC samples of all degrees of differentiation, in 30 OL samples of all degrees of dysplasia, and five normal tissue samples used as the control group. To complete the above procedure, immunohistochemistry was used in a semiquantative manner. The paraffin-embedded tissue samples were selected from the archives of the Department of Oral Medicine/Pathology, School of Dentistry, Aristotle University of Thessaloniki, Greece, from biopsies performed in this department as well as from St. Lukas Hospital of Thessaloniki, Greece, between 2014 and 2019. The study was conducted per the Research and Ethics Committee guidelines of Aristotle University, School of Dentistry, and the Helsinki II declaration (protocol number 29/21.11.2018).

Results

The statistical analysis showed that there was no statistically significant difference in DAPK-1 staining between normal tissues and OLP (p=0.588, independent-samples Kruskal-Wallis test). On the other hand, there was a statistically significant difference between OSCCs and OLPs (p<0,001, independent-samples Kruskal-Wallis test), with the extent of expression of DAPK-1 being greater in OLP than in OSCC. In addition, there was a statistical difference in DAPK-1 expression between OLPs and OLs (p=0,001, independent-samples Kruskal-Wallis test), with DAPK-1 being expressed more in OLP than in OL.

Conclusion

The DAPK-1, as a pro-inflammatory with its role as a tumor suppressor factor, was highly expressed in OLP, both reticular and erosive. The relatively milder expression of DAPK-1 in OL means that, between the two disorders, OLP is less likely to progress to oral cancer. On the other hand, OLP could also offer the background for further investigation of a possible correlation between methylation (immunohistochemical expression of proteins, deriving from potentially DNA-methylated genes) and the development of inflammation. It appears that DNA methylation is important in the regulation of inflammatory genes. Conversely, inflammation may regulate the DNA methylation of many genes involved in carcinogenesis and thus should be taken into account when studying the methylation behavior of genes such as DAPK-1 in pathologies characterized by a sparse inflammatory infiltrate.

## Introduction

Oral lichen planus (OLP) comprises a chronic inflammatory autoimmune disease that is observed in the oral cavity with a frequency of 1% to 2% of patients, with a slight predilection to women of middle and elder age [1]. Clinically, it manifests itself as white papules arranged confluently, drawing a picture of white lines in the form of a network. These lines are widely known as Wicknam's striae and constitute the typical form of the disease, better known as the reticular OLP. Apart from this, the disease can present in other forms, such as the atrophic, bullous, and erosive forms. The erosive and reticular forms comprise the most frequently detected forms of OLP [2]. However, the most important information about OLP and especially its erosive form is a certain capacity of malignant transformation to oral squamous cell carcinoma (OSCC).

International data reveal an overall probability of malignant transformation of OLP ranging from 0.5% to 1.2% [3], while the maximum value that has been reported is 14.3% [4], with high percentages (6.22%) being mentioned in other studies as well [5]. Among the number of factors that affect this probability are the presence of dysplasia [6], smoking, alcohol consumption, or the localization of the lesions, with tongue lesions being the most prone to malignant transformation as well as the infection of hepatitis C virus (HCV) [7]. Besides these, chronic inflammation, apoptosis, and autophagy, the presence of proteins that inhibit the several stages of the cell cycle (p53, Bcl-2, CDKN1A, p16, CDK4 BAX, nuclear factor kappa beta (NF-κB)), especially genetic and epigenetic changes, seem to play a critical role in the process of the transition to malignancy [8].

Among such kinds of genes, DAPK-1 seems to play a crucial role in carcinogenesis since it participates in processes engaged in the carcinogenesis paths and their disturbances by its silencing through hypermethylation. Such kinds of processes are autophagy, apoptosis, and inflammation [9]. The kinase mediates inflammation by contributing to the production of IL-1b. However, it also presents anti-inflammatory properties in pathologies such as ulcerative colitis [10]. Although mutations of its gene are very rare, epigenetic changes affecting it are quite often and are detected very frequently in OSCC.

This very last observation determines the aim of this study. More specifically, the study focuses on the investigation of the epithelial presence of DAPK-1 by means of immunohistochemistry in OLP compared to OSCC, oral leukoplakia (OL), a well-established oral potentially malignant disorder (OPMD), and normal oral epithelium to evaluate its possible role as an early predictor of the possibility of malignant transformation risk of OLP lesions. In our previous work, we aimed to immunohistochemically detect the presence of DAPK-1 in vessels underlying potentially malignant disorders and oral cancer. The lack of OLPs (only six samples) in our previous study was corrected, and in the present study we included 18 OLPs [11]. The statistically nonsignificant results of our previous study may be interpreted in two different ways: either the sample size did not suffice and therefore a statistically significant correlation could not be established, or the vascular endothelial expression of DAPK remains relatively the same in normal, OL, OLP, and OSCC cases and therefore cannot serve as a prognostic factor in oral lesions [11].

## Materials and methods

Immunohistochemistry

To monitor the expression profile of the tumor suppressor protein DAPK-1, immunohistochemical detection took place in various lesions of OLP (reticular and erosive type), in OSCCs of all degrees of differentiation, in OLs of all degrees of dysplasia, and normal tissue samples used as the control group. To complete the above procedure, immunohistochemistry was used in a semiquantative manner. The paraffin-embedded tissue samples were selected from the archives of the Department of Oral Medicine/Pathology, School of Dentistry, Aristotle University of Thessaloniki, Greece, from biopsies performed in this department as well as from St. Lukas Hospital of Thessaloniki, Greece, between 2014 and 2019. The study was conducted per the Research and Ethics Committee guidelines of Aristotle University, School of Dentistry, and the Helsinki II declaration. Approval for the present study was granted by the Ethics Committee of the School of Dentistry, Aristotle University of Thessaloniki, Greece (protocol no. 29/21.11.2018). The inclusion criterion for the study was the presence of sufficient precancerous or cancerous tissue, whereas the exclusion criterion was the presence of inadequate tissue. Parameters such as age, gender, and smoking or alcohol consumption were similar among the various groups of patients. 

For the immunohistochemical procedure, 4 μm-thick sections were picked up from 18 samples of OLP (nine of the erosive type and nine of the reticular type), as shown in Table 1; 22 samples of OSCCs (subdivided into three groups, namely well-differentiated (five samples), moderately differentiated (16 samples), and poorly differentiated (one sample)), as shown in Table 2; 30 samples of OLs (subdivided into three groups: non-dysplastic (10 samples), mildly dysplastic (10 samples), and moderately and severely dysplastic (10 samples)), as shown in Table 3; and five samples of normal oral tissue, which were excised adjacent to fibromas. 

**Table 1:** Samples of OLP and epidemiological characteristics of the patients from whom the samples were derived OLP: Oral lichen planus

OLP form	Localization	Gender	Age (in years)
OLP reticular	Buccal mucosa	M	61
OLP reticular	Buccal mucosa	M	46
OLP reticular	Tongue	M	70
OLP reticular	Tongue	M	57
OLP reticular	Tongue	F	50
OLP reticular	Tongue	F	57
OLP reticular	Buccal mucosa	M	73
OLP reticular	Buccal mucosa	F	49
OLP reticular	Buccal mucosa	F	42
OLP erosive	Tongue	M	63
OLP erosive	Gingiva	M	63
OLP erosive	Gingiva	F	50
OLP erosive	Gingiva	F	77
OLP erosive	Tongue	F	59
OLP erosive	Buccal mucosa	F	58
OLP erosive	Buccal mucosa	F	64
OLP erosive	Buccal mucosa	M	56
OLP erosive	Gingiva	F	26

**Table 2:** Samples of OSCC and epidemiological characteristics of the patients from whom the samples were derived OSCC: Oral squamous cell carcinoma, PD: Poorly differentiated, MD: Moderately differentiated, WD: Well-differentiated

Degree of differentiation	Localization	Gender	Age (in years)
PD	Alveolar process	M	80
PD	Palate	M	35
PD	Lower jaw	M	80
MD	Alveolar process	M	62
MD	Alveolar process	F	56
MD	Alveolar process	F	59
MD	Tongue	F	67
MD	Alveolar process	F	75
MD	Tongue	F	65
MD	Tongue	F	27
MD	Tongue	F	79
MD	Lip	F	77
MD	Floor of the mouth	M	46
MD	Alveolar process	M	72
MD	Tongue	F	42
MD	Buccal mucosa	M	59
MD	Lower jaw	M	30
MD	Tongue	F	82
MD	Gingica	F	78
MD	Gingiva	M	57
WD	Buccal mucosa	F	79
WD	Alveolar process	F	72

**Table 3:** Samples of OL and epidemiological characteristics of the patients from whom the samples were derived OL: Oral leukoplakia, NONDOL: Non dysplastic oral leukoplakia, MDOL: Mildly dysplastic oral leukoplakia, MSDOL: Moderately and severely dysplastic oral leukoplakia

Degree of differentiation	Localization	Gender	Age (in years)
NONDOL	Tongue	M	69
NONDOL	Tongue	M	53
NONDOL	Buccal mucosa	F	58
NONDOL	Buccal mucosa	F	55
NONDOL	Buccal mucosa	F	61
NONDOL	Palate	M	39
NONDOL	Buccal mucosa	M	51
NONDOL	Gingiva	F	60
NONDOL	Buccal mucosa	F	58
NONDOL	Buccal mucosa	M	55
MDOL	Buccal mucosa	M	55
MDOL	Gingiva	F	60
MDOL	Buccal mucosa	F	58
MDOL	Tongue	F	41
MDOL	Buccal mucosa	M	51
MDOL	Buccal mucosa	F	58
MDOL	Tongue	M	69
MDOL	Buccal mucosa	F	61
MDOL	Buccal mucosa	M	56
MDOL	Buccal mucosa	M	69
MSDOL	Tongue	F	65
MSDOL	Tongue	M	57
MSDOL	Gingiva	F	62
MSDOL	Tongue	F	70
MSDOL	Gingiva	F	70
MSDOL	Floor of the mouth	M	69
MSDOL	Tongue	M	52
MSDOL	Tongue	M	64
MSDOL	Tongue	F	70
MSDOL	Tongue	M	52

The conventional histological staining of hematoxylin and eosin (H&E) was used to confirm the initial diagnosis for the whole number of specimens. The immunohistochemical experiment aimed to detect the expression of the anti-DAPK-1 (Novus Biologicals, Centennial, CO, USA) diluted in 1:100. The secondary system of staining detection, Dako Envision Flex+ (Dako, Glostrup, DNK), was used following the manufacturer’s instructions. The staining pattern was indicated by the use of the chromogen (Dako Dab Envision Chromogen). The overall immunohistochemical staining evaluation was fulfilled by the microscopic examination of the tissue specimens and recording of the corresponding results. The evaluation was completed by calculating the percentage of positively stained epithelial cells on a scale of 1 to 4 (Table 4). The staining was deemed positive when the cytoplasm, the nucleus, or the membrane turned brown.

**Table 4:** Scale evaluating the range of percentage of stained epithelial cells

Grade of staining	Range of percentage of stained cells
0	< 5% of cells were stained
I	6% to 25% of cells were stained
II	26% to 50% of cells were stained and
III	≥ 50% of cells were stained

Statistical analysis

The statistical analysis was performed using SPSS Statistics version 29 (IBM Corp., Armonk, NY, USA). The independent-samples Kruskal-Wallis test was used for the statistical analysis of the immunohistochemical experiment and the comparison between OLP vs. OSCC, OLP vs. OL, and OLP vs. normal. A two-sided p-value < 0.05 was considered as statistically significant.

## Results

The descriptive analysis revealed that in 80% (4/5) of normal tissue samples, a very strong expression of DAPK-1 was observed (grade III). A similar pattern of staining intensity was detected in OLP lesions in which maximum staining intensity (grade III) showed up in the total number of lesions (18/18), i.e., 100%, whereas in OSCC lesions the expression of DAPK-1 was mostly mild (grade I) at 54.54% (12/22) of the lesions, moderate (grade II) in 36.36% (8/22) of OSCCs, and only 16.66% (2/22) of the samples were characterized by an intense expression (grade III). In OL lesions, the expression of DAPK-1 was intense in 43.33% (13/30) of the lesions (grade III), moderate in 30% (9/30) of the lesions (grade II), and mild in 26.67% (8/30) of the lesions (grade I) (Table 5).

**Table 5:** Distribution of DAPK-1 immunohistochemical expression among lesions of OLP, OSCCs, OLs, and normal tissues OLP: Oral lichen planus, OSCC: Oral squamous cell carcinoma, OL: Oral leukoplakia

Variables			Staining grade			Total
			I'	II'	III'	
Type of lesion	Normal tissues		0	1	4	5
	OSCC		12	8	2	22
	OLP		0	0	18	18
	OL		8	9	13	30
Total			20	18	37	75

Moreover, statistical analysis observed that there was no statistically significant difference in DAPK-1 staining between normal tissues and OLP (p=0.588, independent-samples Kruskal-Wallis test). On the other hand, there was a great statistical difference between OSCCs and OLP lesions (p<0.001, independent-samples Kruskal-Wallis test), with the extent of expression of DAPK-1 being greater in OLP than in OSCCs. In addition, there was a statistical difference in DAPK-1 expression between OLPs and OLs (p=0.001, independent-samples Kruskal-Wallis test) as expected, with DAPK-1 being expressed in a far more intense way in OLP than in OL (Table 6).

**Table 6:** Statistical comparison of the different levels of DAPK-1 expression in OSCC vs. OLP, OL vs. OLP, and normal vs. OLP OSCC: Oral squamous cell carcinoma, OLP: Oral lichen planus, OL: Oral leukoplakia

Comparison of samples	p-value
OSCC vs. OLP	p<0.001, independent-samples Kruskal-Wallis test
OL vs. OLP	p=0.001, independent-samples Kruskal-Wallis test
NORMAL vs. OLP	p=0.588, independent-samples Kruskal-Wallis test

**Figure 1:**
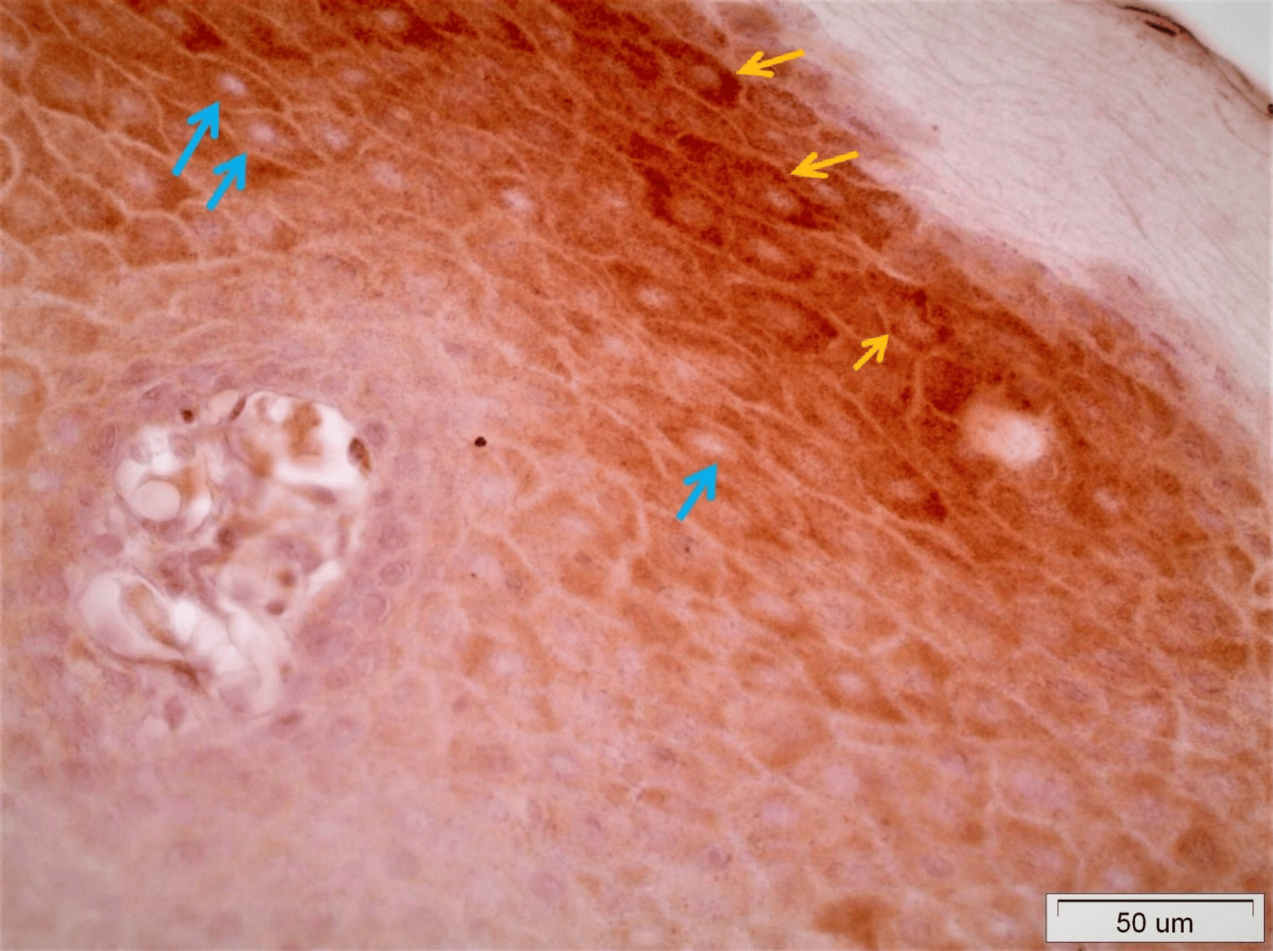
DAPK-1 staining in normal epithelium Seen is an intense cytoplasmic staining mostly in cells of the spinous layer (blue arrows) and a nuclear one in several cells of the upper third of the spinous and granular layer (yellow arrows) (x40).

On observing the staining motifs of the samples from normal tissue, OLP, and OSCC, several interesting details came into consideration. As far as normal tissue is concerned, DAPK-1 had a highly cytoplasmic expression in epithelial cells, mostly of the spinous and, to a lesser extent, the granular layer. However, some cells with nuclear DAPK-1 staining were also detected (Figure 1).

**Figure 2:**
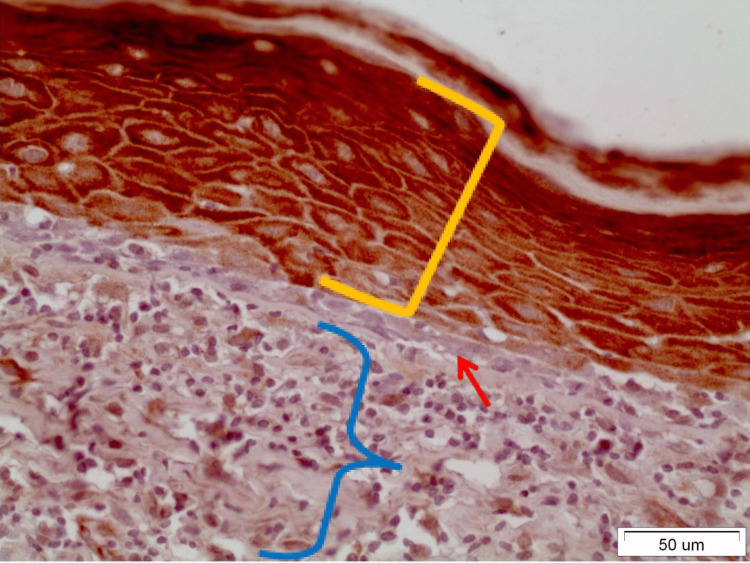
Moderate inflammatory lymphocytic infiltration (blue bracket) of the underlying connective tissue is observed in a reticular OLP lesion. At the same time, intense staining of all layers of the epithelium (yellow bracket) except the basement membrane (red arrow) is observed. Staining remains cytoplasmic and membranous and rarely nuclear (x40). OLP: Oral lichen planus

**Figure 3:**
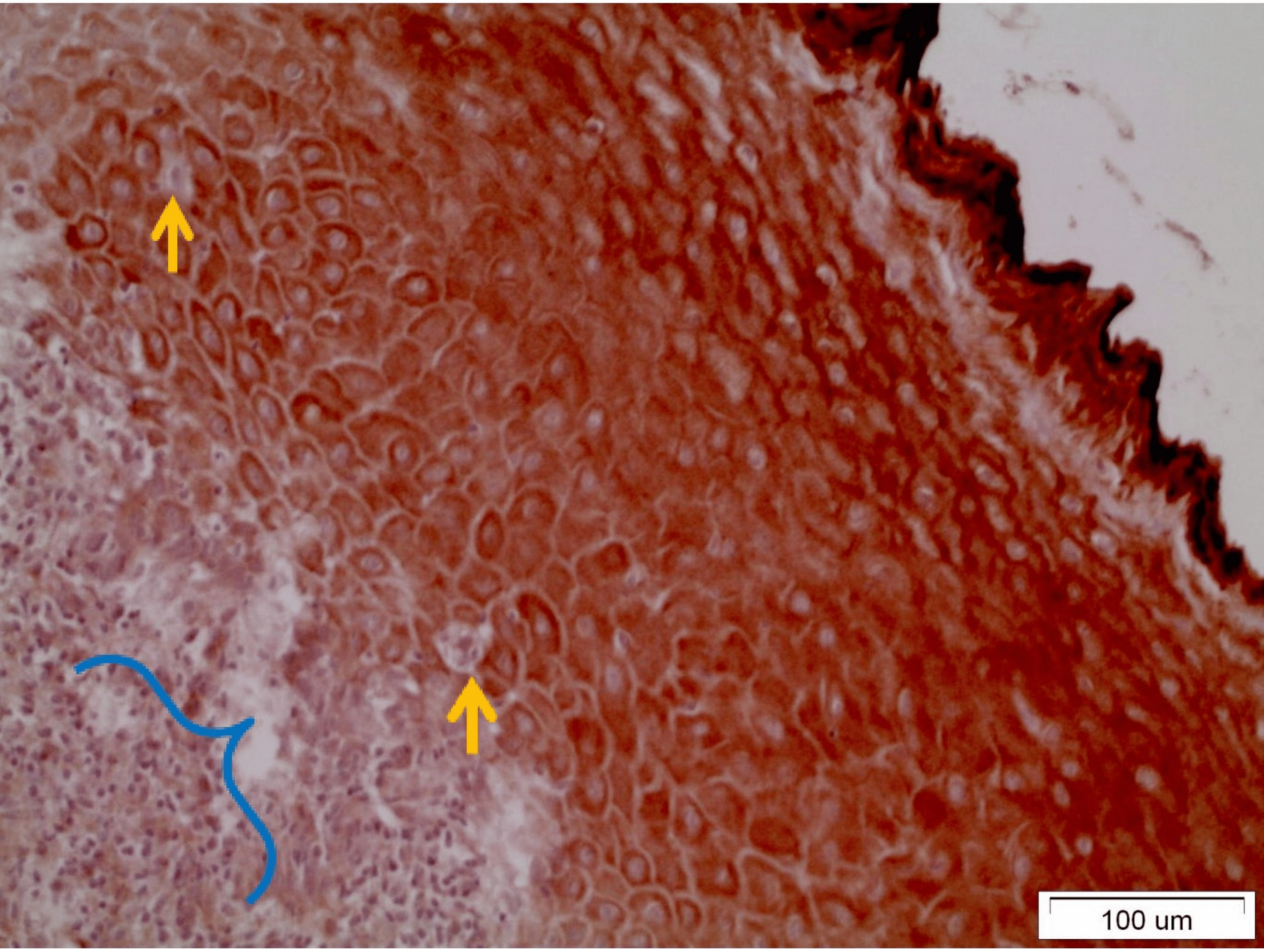
Seen is an intense cytoplasmic and membranous staining of all layers of the epithelium of an erosive OLP lesion combined with intense inflammatory lymphocytic infiltration (blue bracket) in the underlying dermis. Also observed are colloidal apoptotic bodies of civatte in both the basal and suprabasal layers (yellow arrows) (x20). OLP: Oral lichen planus

**Figure 4:**
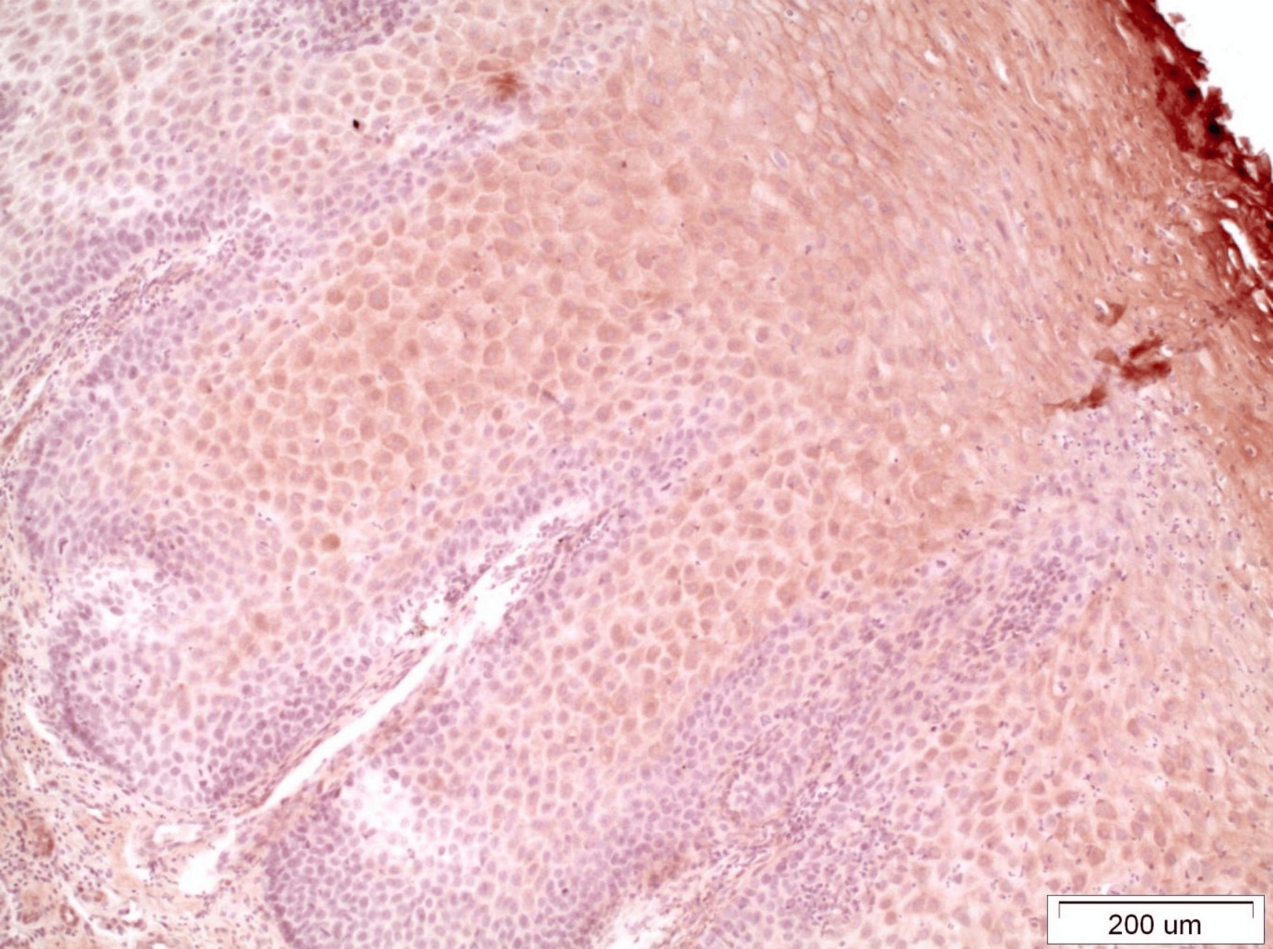
This is an example of severely dysplastic OL, which was relatively better stained, but still, the expression of DAPK-1 remains mild and diffuse (x10). OL: Oral leukoplakia

In OLP lesions where the expression of DAPK-1 is quite strong (grade III), both in the reticular and erosive forms, intense staining was observed in most layers of the epithelium except the basal-suprabasal. The staining was mainly cytoplasmic and membranous (Figure 2). It was interesting to note that in areas of denser and extended lymphocytic infiltration in the underlying connective tissue, the expression of DAPK-1 was more intense in the epithelium (Figure 3). In OL lesions, particularly of severe dysplasia, the expression of DAPK-1 was relatively mild and weak (Figure 4).

**Figure 5:**
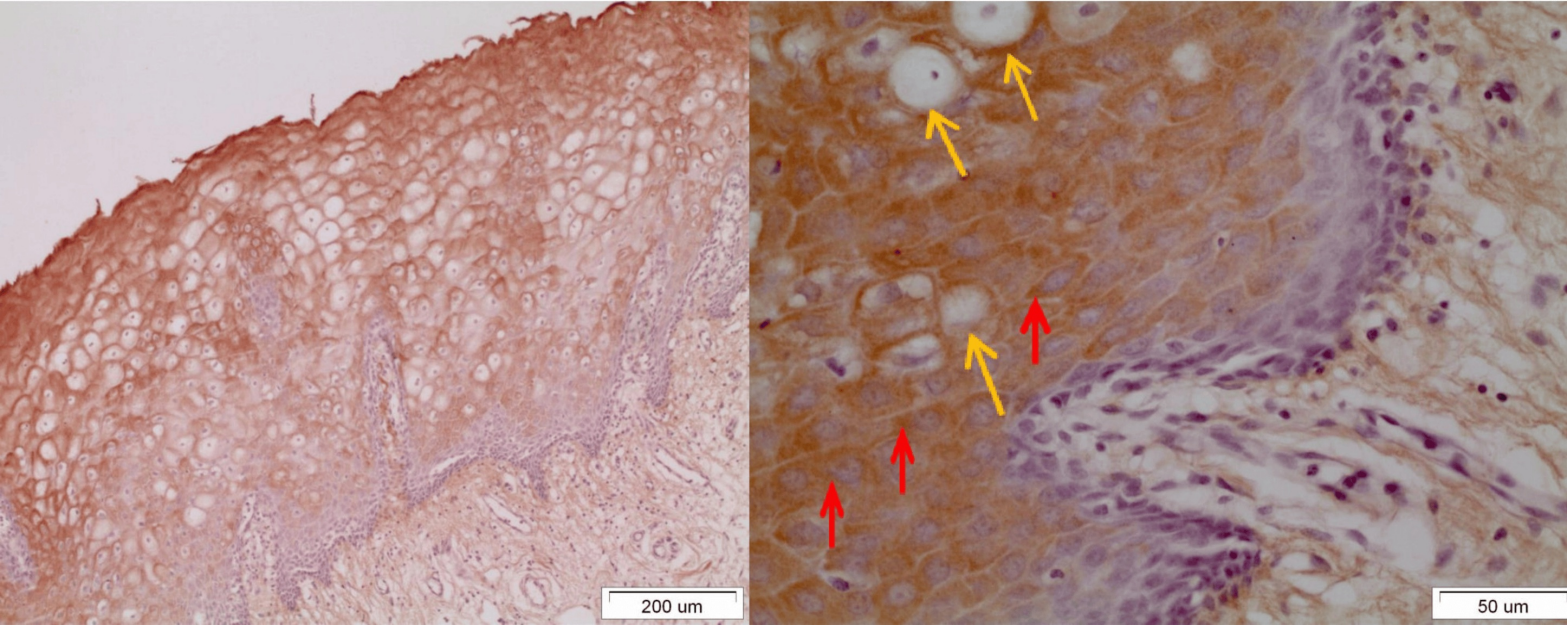
DAPK-1 staining in a low differentiated OSCC lesion A: Staining seems to be intense in all layers of the epithelium except the basal and suprabasal (x10); B: The expression of DAPK-1 is observed to be mainly cytoplasmic (red arrows) as well as membranous (yellow arrow) (x40) OSCC: Oral squamous cell carcinoma

In OSCCs, the overall expression of DAPK-1 was weak in the majority of samples (grade I). This was not valid for the low-differentiated carcinomas where DAPK-1 seems to be highly expressed (Figure 5).

**Figure 6:**
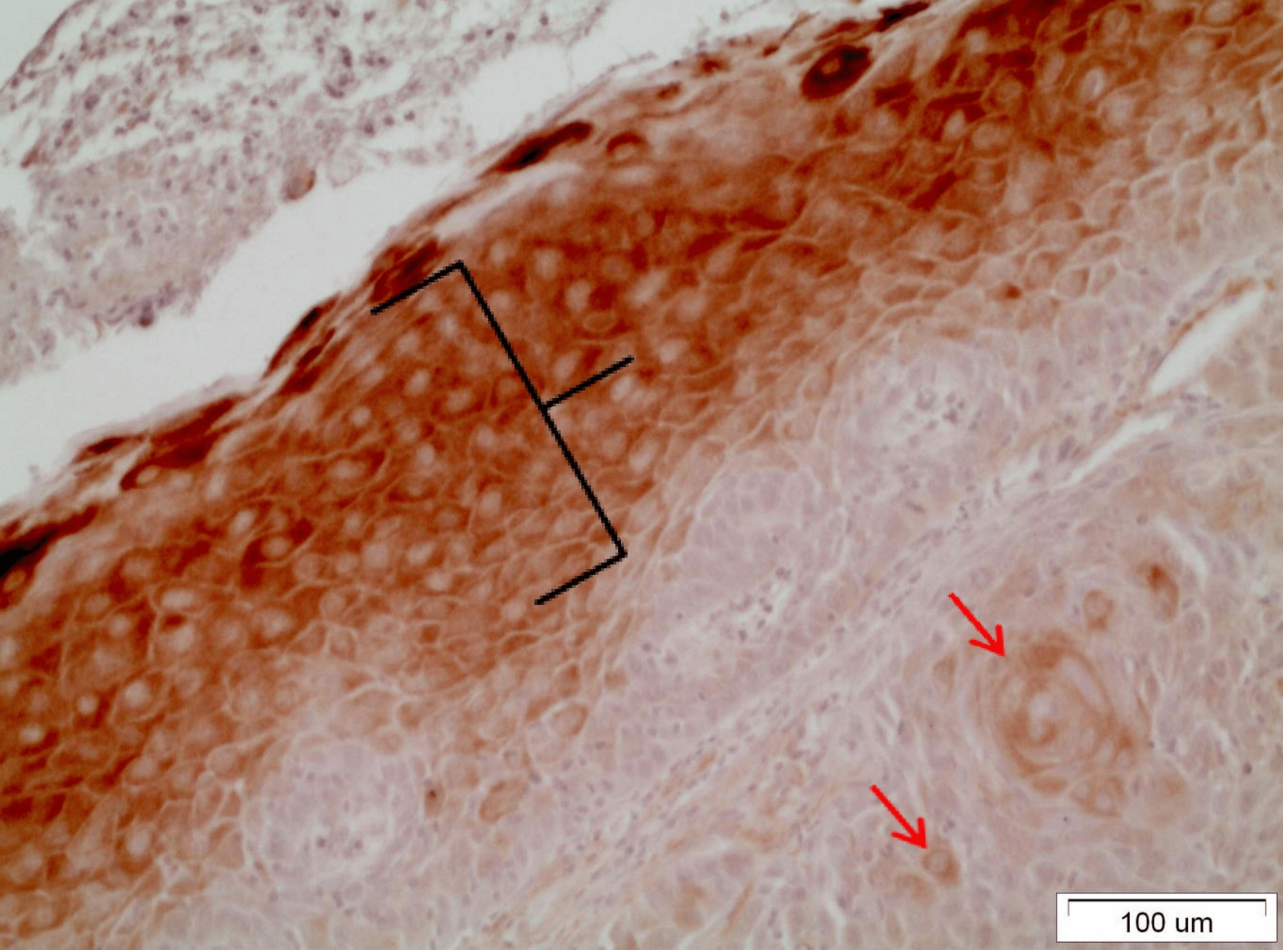
Distinct sporadic presence of DAPK-1 in epithelial neoplastic islands (red arrow) in moderately differentiated OSCC. The DAPK-1 is expressed in the spinous and granular layer (black bracket), of the epithelium (x20). OSCC: Oral squamous cell carcinoma

It is noteworthy that DAPK-1 is highly expressed in the very center of the epithelial islands as well as adjacent to the keratin pearls. In addition, the antibody of the kinase is intensely detected in the spinous and granular layer of the epithelium neighboring the neoplastic foci, whether it shows dysplasia or not (Figure 6).

**Figure 7:**
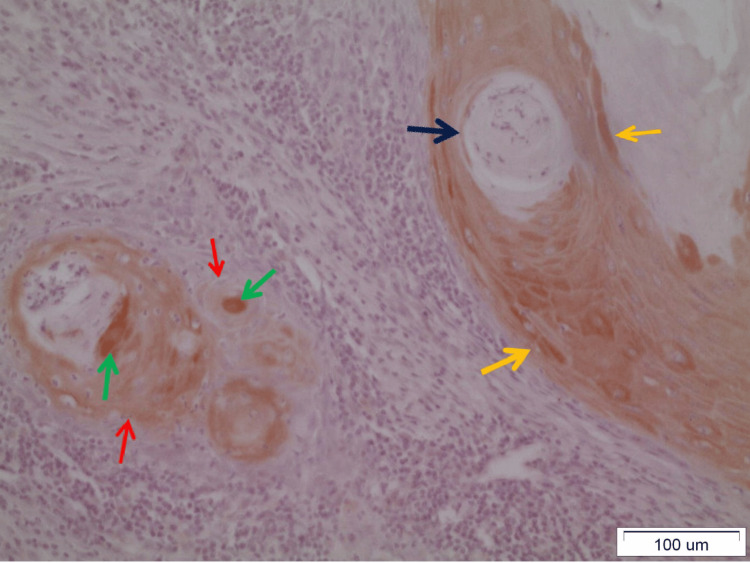
Keratin pearl (dark blue arrow) in a well-differentiated OSCC surrounded by DAPK-1 highly-stained neoplastic epithelial cells. In the center of neighboring neoplastic epithelial islands, the staining is intense (green arrows) whereas it gradually turns milder in the periphery of the islands (red arrows). Staining is mainly cytoplasmic and, in some cases, nuclear (yellow arrows) (x40). OSCC: Oral squamous cell carcinoma

This motif of expression of DAPK-1 in the apparently normal epithelium adjacent to the tumor site is more prominent mainly in cases of moderately and well-differentiated carcinomas. Staining in most cases is cytoplasmic, and only in rare cases does it seem to be nuclear or, even more rarely, membranous too (Figure 7).

## Discussion

Oral cancer is a major problem since it affects approximately 450,000 people annually, causing 350,000 deaths, which ranks it as the 18th most common cancer worldwide and the 8th and 15th most common for men and women in the USA, respectively [12,13]. Oral potentially malignant disorders (OPMDs) may lead to oral cancer, with OL being the most common OPMD and OLP (especially its erosive form) being a rarer form as it is characterized by the potential of malignant transformation ranging from 1% to 6% [14]. The need for early prognostic prediction of this potential for malignant transformation of OLP lesions led the research towards cancer stem cell biomarkers and, in our study, DAPK-1. 

The DNA methylation may inhibit the function of various genes (mainly tumor suppressors) and thus promote carcinogenesis [15]. The main property of DAPK-1 is its tumor-suppressing ability, which in many malignancies, including OSCC, is inhibited through epigenetic changes, such as the aforementioned DNA methylation [16].

Additionally, DAPK-1 takes part in a series of processes such as apoptosis and autophagy in response to stress conditions of the endoplasmic reticulum of epithelial cells. It also contributes to the inhibition of necrosis through its connection with p38 MAPK-2 and its subsequent activation [9]. In addition, it promotes inflammatory processes through its participation in signaling activated by pro-inflammatory molecules such as IFN-γ tumor necrosis factor, TNF-α, or TTGF-β, etc., as well as in processes that lead to the production of cytokines such as IL-1b and IL-18. At the same time, it exhibits an anti-inflammatory effect mainly through the attenuation of the activation of T-lymphocytes through the selective inhibition of their receptors, which is triggered by the activation of the transcription factor NF-κB [17].

To the best of our knowledge, there is no literature on the immunohistochemical expression of DAPK-1 in OLP lesions. Immunohistochemistry may designate, in contrast to other techniques such as flow cytometry or polymerase chain reaction (PCR), the number of cells expressing DAPK-1 throughout the epithelium of the examined section obtained from paraffin-embedded tissues. It is able, in other words, to determine which parts of cellular populations are stained positive and thus express DAPK-1 and their localization throughout the epithelium. In this way, it is a semiquantitative method evaluating at the same time qualitative characteristics as the intensity of staining, which represents the presence of the kinase.

In a study by Jana et al., in 25 samples of OLP erosive and 25 of the reticular form, it was found that erosive lichen planus is subjected to more intense oxidative stress caused by active oxygen radicals [18]. The production of nitric oxide (NO), which is a highly reactive free radical, was increased in the erosive lichen lesions compared to the reticular ones. An inflammatory substrate, which in the erosive form is clearly more intense, has the ability to promote DNA mutagenesis, which favors malignant transformation. In the same study, higher levels of apoptosis were detected in the erosive OLP lesions than in reticular ones. This result was also confirmed by the study by Brant et al. in 2012, in which higher levels of apoptosis were detected in lesions of erosive lichen compared to those of the reticular form [19].

In the present study, the expression of DAPK-1, which promotes apoptosis, in the lesions mainly of the erosive lichen is more intense in the epithelium; the denser the inflammatory infiltrate, the greater the number of lymphocytes. An increased number of civatte bodies was identified in the lesions of erosive OLP in relation to those of the reticular form, while the immunohistochemical expression of the kinase is not limited to the basal and parabasal layer but becomes more intense in the other epithelial layers. This picture is prominent in the erosive lesions and contrasts with the study by Brant et al. (2008), in which the expression of the kinase is more intense in the basal and parabasal layers, which are more vulnerable to the aggressiveness of the inflammatory infiltrate [20]. However, the presence of dense and extensive inflammation is common in both studies.

There is an interesting issue that could call for a different role of DAPK-1. The fact that its expression increases when inflammation increases makes it a potential link between apoptosis and inflammation. The persistence of chronic inflammation in turn has been widely related to the presence of malignant transformation and therefore carcinogenesis [21]. Thus, a valuable hypothesis arises according to which DAPK-1, due to its apoptotic and tumor-suppressing abilities in both its active and methylated inactive form, could potentially be used as an early predictor of the possibility of malignant transformation of an OLP lesion through the study of its correlation with the inflammation that characterizes the OLP lesions.

Chronic inflammation increases the probability of malignant transformation, and in particular, inflammatory cytokines such as IL-6, IL-17, IL-23, and TNF-α and TGF-β are released by T-lymphocytes and cause fundamental changes in the proteins of the epithelium contributing to malignant transformation [22]. Likewise, DAPK-1 contributes to the transmission of inflammatory signaling and subsequently inflammation. After all, DAPK-1 was initially identified due to its role in apoptosis through its activation by IFN-γ, but also its involvement in apoptotic processes triggered by the contribution of TNF-α, TGF-β, and other inflammatory factors [17].

In our study, the OLP samples expressed DAPK-1 in a very intense manner, signifying that both its tumor-suppressing function and its pro-inflammatory role are present. The DAPK-1 was expressed statistically significantly lower in OL, thus signifying a relative loss of expression and a possible loss of its tumor-suppressing ability in OL lesions. Therefore, compared to OL, OLP remains a more benign OPMD. At the same time, OLP expressed statistically significantly more DAPK-1 than OSCC, which makes sense since in cancerous lesions, tumor-suppressing genes are expected to be silenced. However, there are some limitations to our study, namely the lack of tumor, nodes, and metastases (TNM) classification, the five-year survival rate of the OSCC cases, and the lack of information regarding the clinical course of the potentially malignant disorders, OL and OLP. 

## Conclusions

Due to its pro-inflammatory nature and role as a tumor suppressor factor, DAPK-1 is highly expressed in OLP (both reticular and erosive types). The relatively milder expression of DAPK-1 in OL means that, between the two disorders, OLP is less likely to progress to oral cancer. On the other hand, OLP could also offer the background for further investigation of a possible correlation between methylation (immunohistochemical expression of proteins deriving from potentially DNA-methylated genes) and the development of inflammation. It appears that DNA methylation is important in the regulation of inflammatory genes. Conversely, inflammation may regulate the DNA methylation of many genes involved in carcinogenesis and thus should be taken into account when studying the methylation behavior of genes such as DAPK-1 in pathologies characterized by a sparse inflammatory infiltrate.
